# Prevalence, trends, and outcomes of atrial fibrillation in hospitalized patients with metastatic cancer: findings from a national sample

**DOI:** 10.1002/cam4.4105

**Published:** 2021-07-07

**Authors:** Hedong Han, Longpei Chen, Zhen Lin, Xin Wei, Wei Guo, Yamei Yu, Cheng Wu, Yang Cao, Jia He

**Affiliations:** ^1^ Department of Respiratory and Critical Care Medicine Jinling Hospital Nanjing University School of Medicine Nanjing China; ^2^ Department of Health Statistics Second Military Medical University Shanghai China; ^3^ Department of Medical Oncology Shanghai Changhai Hospital Second Military Medical University Shanghai China; ^4^ Department of Cardiology Virginia Commonwealth University Richmond VA USA; ^5^ Department of Medicine Mount Sinai St. Luke’s and West Medical Center New York NY USA; ^6^ Department of Cardiology Shanghai Changning District Central Hospital Shanghai China; ^7^ Clinical Epidemiology and Biostatistics School of Medical Sciences Örebro University Örebro Sweden; ^8^ Department of Health Statistics Tongji University School of Medicine Shanghai China

**Keywords:** atrial fibrillation, cancer, outcomes, prevalence, trends

## Abstract

**Background:**

Epidemiological evidence regarding the link between cancer and atrial fibrillation (AF) are limited and outcomes of metastatic cancer comorbid with AF need to be elucidated.

**Objective:**

This study aims to evaluate the prevalence, temporal trends, and outcomes of AF in hospitalized metastatic cancer patients.

**Methods:**

The National Inpatient Sample (NIS) database was used to identify adult patients with metastatic tumors from 2003 to 2014. We analyzed the trends in AF prevalence, in‐hospital mortality, total cost, length of stay (LOS), and comorbidities pertaining to metastatic cancer. Multivariable‐adjusted models were used to evaluate the association of AF with clinical factors, in‐hospital mortality, total cost, and LOS.

**Results:**

Among 2,478,598 patients with metastatic cancer, 8.74% (216,737) were diagnosed with AF. The proportion of comorbid AF increased from 8.28% in 2003 to 10.06% in 2014 (*p* < 0.0001). Older age, white race, male, Medicare, higher income, larger hospital bed size, and urban teaching hospital were associated with higher AF occurrence. Among primary tumor sites, lung cancer experienced the highest odds of AF compared to other cancers. Patients with metastasis to lymph node and respiratory organ had higher odds of AF. In metastatic cancer, AF was associated with higher in‐hospital mortality (odds ratio: 1.48; 95% confidence interval: 1.43–1.54), 18% longer LOS, and 19% higher cost.

**Conclusions:**

AF prevalence in metastatic cancer continues to increase from 2003 to 2014. AF is linked to poorer prognosis and higher healthcare resource utilization. As the population ages, optimal preventive and treatment management strategies are needed for metastatic cancer comorbid with AF.

## Graphical abstract

Atrial fibrillation (AF) prevalence in metastatic cancer continues to increase from 2003 to 2014. AF is linked to poorer prognosis and higher healthcare resource utilization. As the population ages, optimal preventive and treatment management strategies are needed for metastatic cancer comorbid with AF.

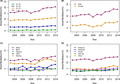

## 1 INTRODUCTION

Atrial fibrillation (AF) is the most common arrhythmia and almost 2%–3% of the general population in the United States (US) suffer from AF. An estimate of 2.7–6.1 million U.S. people in 2010 have AF, which is predicted to more than double by 2030.^1^ According to the trend analysis of the Framingham Heart Study, the adjusted AF prevalence has increased fourfold from 1958 to 2007 over a period of 50 years of observation.^2^ AF is associated with increased risk of death, renal disease, and cardiovascular events such as stroke or heart failure.^3^

Cancer is the second cause of death and approximately 1,762,450 new cancer cases and 606,880 cancer deaths are projected to occur in the United States in 2019.^4^ Metastases account for a majority of cancer‐related deaths.^5^ Recent data indicated an increased incidence and prevalence of AF in cancer patients.^6^ The occurrence of AF in cancer may be attributable to direct tumor effect or comorbid conditions or cancer treatments such as surgery or medications.^7^, ^8^ In particular, post‐operative AF is the most common form of cancer‐associated AF.^9^, ^10^ Previous studies focusing on several types of cancer have suggested the increased AF risk following surgery, especially pulmonary resection for lung cancer.^9^ In a large cohort study, AF was post‐operatively present in as high as 12.6% of patients who underwent lung cancer surgery.^11^ Anticoagulant therapy for AF in cancer posed physicians a challenge due to the possibility of both increased risk of thrombus and bleeding.^9^, ^10^ Current guidelines do not address the issue of anticoagulation for AF in cancer patients and existing randomized clinical trials of anticoagulants for the prevention of stroke routinely excluded patients with cancer, particularly metastatic cancer.^12^ Optimal preventive and treatment management strategies targeting this specific population are not well‐established.

The increased incidence and prevalence of AF among patients with cancer would be of considerable public health importance. However, epidemiological evidence with regards to the link between cancer and AF are limited and outcomes of metastatic cancer comorbid with AF need to be elucidated.^8^, ^9^, ^10^ This study aims to (1) describe temporal trends in the prevalence of AF in hospitalized metastatic cancer patients in the United States from 2003 to 2014; (2) evaluate the association between demographic, hospital‐related characteristics, tumor‐related characteristics, and AF occurrence; (3) examine the impact of AF on in‐hospital mortality, hospital cost, and length of stay (LOS) among patients with metastatic cancer.

## 2 MATERIALS AND METHODS

### 2.1 Data source

National Inpatient Sample (NIS) is the largest all‐payer care database in the United States, which provided the Healthcare Cost and Utilization Project. Before 2012, the database was constructed by sampling 20% of U.S. community hospitals. In 2012, the NIS underwent a redesign and was drawn as a 20% stratified systematic sample of discharges from all participating hospitals, which could decrease the sampling error and make estimates more generalizable to the target population.

### 2.2 Study population

We identified adult patients (aged ≥18 years) with a primary diagnosis of cancer among the 10 most common solid‐organ malignancies in the United States^13^ using the International Classification of Diseases, Ninth Revision, Clinical Modification (ICD‐9‐CM) codes for cancer of lung, stomach, pancreas, colon/rectum, prostate, bladder, breast, endometrium, ovary, and kidney, along with secondary diagnostic codes for metastasis (Supplemental eTable 1).^14^ Metastatic sites for evaluation included bone & bone marrow, brain & spinal cord, lymph nodes, liver, respiratory organs, urinary organs, adrenal glands, gastrointestinal organs (other than liver), genital organs, and other organs.

### 2.3 Covariate assessment

The NIS contains data on patient‐level and hospital‐level characteristics for each admission record. Demographic characteristics included age, gender, race, type of insurance, and income. Hospital characteristics included hospital bed size, type, and region. Comorbidities were classified using the Elixhauser comorbidity index (ECI) score which included 29 comorbidities to represent the severity of comorbid conditions (excluded cancer).^15^ We further extracted additional information regarding coronary artery disease, prior stroke, chemotherapy, CHA_2_DS_2_‐VASc score, and long‐term anticoagulants that were not included in the ECI score. Major diagnostic or therapeutic operating room procedures based on the principal Clinical Classifications Software procedure codes related to the same admission were considered.

### 2.4 Primary and secondary outcomes

The primary outcome was the temporal trend of AF prevalence among patients with metastatic cancer. AF was defined through secondary diagnoses fields using ICD‐9‐CM code 427.31. This established methodology for AF ascertainment has been previously used and validated with high specificity (98%) and positive predictive value (89%).^16^ The secondary outcomes included AF predictors, in‐hospital mortality, LOS, and total cost. We used the cost‐to‐charge ratio to adjust total charges to the total cost and then used the consumer price index to account for inflation.

### 2.5 Statistical analysis

Using the NIS sampling and weighting strategy, we established national estimates for all measures in the analyses. Demographic, hospital, and tumor‐level characteristics were expressed with proportions and compared using χ^2^ test. Cochran–Armitage trend test was used to assess the trends of AF prevalence (categorized by age, gender, race, insurance type or primary tumor site) and in‐hospital mortality. Cuzick nonparametric test was used to detect the trend of LOS and total cost over time.^17^

We performed univariable logistic model (model 1) and two multivariable‐adjusted logistic models (models 2 and 3) to assess the risk predictors of AF. Adjustments in model 2 included demographics, hospital‐level factors, tumor‐related factors (primary tumor sites, metastatic sites, and the number of metastatic sites), major operating room procedure, chemotherapy, long‐term anticoagulants, ECI score, and CHA_2_DS_2_‐VASc score. Adjustments in model 3 were demographics, hospital‐level factors, tumor‐related factors, major operating room procedure, chemotherapy, long‐term anticoagulants, individual ECI comorbidities, coronary artery disease, and prior stroke.

We used two multivariable‐adjusted models (models a and b) to evaluate the association of AF with LOS, cost, and in‐hospital mortality. Model a was adjusted for demographics, hospital‐level factors, tumor‐related factors, and ECI score. In model b, we replaced the ECI score with individual comorbidities. Because the distributions of LOS and cost are skewed, we made log‐transformations before multivariable analysis. Subgroup analyses were further conducted according to various patient characteristics. We also evaluated trends in in‐hospital mortality, LOS, and cost in patients with and without comorbid AF treating year as a continuous variable.

The amount of missing data was less than 1% for most variables, except for race (17.13%), cost (6.45%), and income (2.25%; Supplemental eTable 2). Missing race was regarded as the missing group. Missing data of other categorical variables were imputed with the dominant category. Sensitivity analyses were conducted in the following alternative settings: (1) unweighted analysis; (2) complete case analysis with the exclusion of all missing records; (3) exclusion of patients receiving major operating room procedure; (4) double robust inverse probability of treatment weighting analysis using the propensity score^18^; (5) multiple imputation to deal with missing data using five imputed datasets with the assumption that the data were missing at random.

All tests were two‐sided and *p*‐values ≤0.05 were considered significant. Statistical analyses were performed using SAS version 9.4 (SAS Institute Inc., Cary, NC).

## 3 RESULTS

### 3.1 Basic characteristics

From 2003 to 2014, this study identified a weighted cohort of 2,478,598 (unweighted 503,060) patients with metastatic cancer. Basic characteristics are displayed in Table 1. Overall, 8.74% (*n* = 216,737) patients had a diagnosis of AF. Patients with AF were older (74.51 vs. 64.59 years), less likely to be female (44.90% vs. 56.53%), more likely to be white (71.79% vs. 59.98%), had higher CHA_2_DS_2_‐VASc score (2.52 vs. 1.39), higher ECI score, and frequent individual comorbidities (Supplemental eTable 3). Among cancer patients with comorbid AF, most had lung cancer (42.02%) as the primary tumor site, followed by colorectal cancer (24.84%) and pancreas cancer (8.20%) and the most common metastatic sites were lymph nodes (38.39%), liver (26.60%), and respiratory organs (20.57%). Multiple metastatic sites occurred in 30.74% of cancer patients with comorbid AF.

**TABLE 1:** Demographics, hospital characteristics, cancer‐related factors, and outcomes of metastatic cancer patients with and without AF

Variables	AF (*N* = 216,737, %)	Without AF (*N* = 2,261,861, %)	*p*‐value
Mean age (SE)	74.51 (0.07)	64.59 (0.09)	<0.0001
Age group			<0.0001
18–44	797 (0.37)	151,370 (6.69)	
45–64	34,416 (15.88)	947,975 (41.91)	
65–74	64,921 (29.95)	601,752 (26.61)	
≥75	116,603 (53.80)	560,764 (24.79)	
Female	97,323 (44.90)	1,276,372 (56.43)	<0.0001
Race			<0.0001
White	155,604 (71.79)	1,356,773 (59.98)	
Black	12,327 (5.69)	254,715 (11.26)	
Hispanic	6,798 (3.14)	142,857 (6.32)	
Other	7,332 (3.38)	116,810 (5.17)	
Missing	34,676 (16.00)	390,706 (17.27)	
Type of insurance			<0.0001
Medicare	166,783 (76.95)	1,102,480 (48.74)	
Medicaid	7,515 (3.47)	222,056 (9.82)	
Private	35,695 (16.47)	783,485 (34.64)	
Self‐pay	2,673 (1.23)	78,290 (3.46)	
Other	4,072 (1.88)	75,551 (3.34)	
Income quartile			<0.0001
Q1	53,446 (24.66)	637,573 (28.19)	
Q2	55,505 (25.61)	564,110 (24.94)	
Q3	53,935 (24.89)	537,191 (23.75)	
Q4	53,851 (24.84)	522,987 (23.12)	
Hospital bed size			0.1389
Small	23,687 (10.93)	248,453 (10.98)	
Medium	50,656 (23.37)	513,621 (22.71)	
Large	142,394 (65.70)	1,499,787 (66.31)	
Hospital type			<0.0001
Rural	21,203 (9.78)	217,659 (9.62)	
Urban non‐teaching	86,647 (39.98)	833,700 (36.86)	
Urban teaching	108,887 (50.24)	1,210,502 (53.52)	
Hospital region			<0.0001
Northeast	51,183 (23.62)	501,539 (22.17)	
Midwest	53,317 (24.60)	513,334 (22.70)	
South	74,932 (34.57)	829,744 (36.68)	
West	37,305 (17.21)	417,244 (18.45)	
Elixhauser comorbidity index			<0.0001
0	11,921 (5.50)	407,519 (18.02)	
1	35,519 (16.39)	543,291 (24.02)	
2	51,049 (23.55)	538,129 (23.79)	
≥3	118,248 (54.56)	772,922 (34.17)	
CHA_2_DS_2_‐VASc (median, IQR)	2.52 (1.44–3.57)	1.39 (0.46–2.57)	<0.0001
Primary tumor site			<0.0001
Lung	91,082 (42.02)	643,636 (28.46)	
Stomach	10,807 (4.99)	116,679 (5.16)	
Pancreas	17,774 (8.20)	199,589 (8.82)	
Colon/rectum	53,848 (24.84)	575,091 (25.42)	
Prostate	7,045 (3.25)	79,387 (3.51)	
Bladder	7,544 (3.48)	66,048 (2.92)	
Breast	10,741 (4.96)	294,559 (13.02)	
Endometrium	3,632 (1.68)	61,454 (2.72)	
Ovary	8,162 (3.77)	149,744 (6.62)	
Kidney	6,104 (2.81)	75,674 (3.35)	
Chemotherapy	9,466 (4.37)	108,479 (4.80)	0.0004
Number of metastatic sites (≥2)	66,625 (30.74)	752,664 (33.28)	<0.0001
Major operating room procedure	118,321 (54.59)	1,301,566 (57.54)	<0.0001
Long‐term anticoagulants	25,168 (11.61)	36,399 (1.61)	<0.0001
Coronary artery disease	51,628 (23.82)	238,528 (10.55)	<0.0001
Prior stroke	10,962 (5.06)	55,662 (2.46)	<0.0001
Metastatic site			
Bone & bone marrow	37,831 (17.45)	381,635 (16.87)	0.0047
Brain & spinal cord	16,682 (7.70)	216,217 (9.56)	<0.0001
Lymph nodes	83,206 (38.39)	891,809 (39.43)	0.0002
Liver	57,661 (26.60)	641,590 (28.37)	<0.0001
Respiratory organs	44,591 (20.57)	400,215 (17.69)	<0.0001
Urinary organs	5,595 (2.58)	62,941 (2.78)	0.0185
Adrenal glands	9,092 (4.19)	84,844 (3.75)	<0.0001
Gastrointestinal organs	25,975 (11.98)	350,031 (15.48)	<0.0001
Genital organs	4,645 (2.14)	84,046 (3.72)	<0.0001
Other organs	22,873 (10.55)	235,877 (10.43)	0.4506
Death	30,550 (14.11)	206,446 (9.13)	<0.0001
LOS (median, IQR)	7.11 (3.91–12.00)	5.08 (2.47–8.81)	<0.0001
Total cost (median, IQR)	18,123 (9,805–31,794)	13,602 (7,797–23,166)	<0.0001

Abbreviations: IQR, interquartile range; LOS, length of stay; Q1, 0–25th Percentile; Q2, 20–50th Percentile; Q3, 50–75th Percentile; Q4, 75–100th Percentile; SE, standard error.

### 3.2 Trends of AF prevalence in metastatic cancer

**FIGURE 1:**
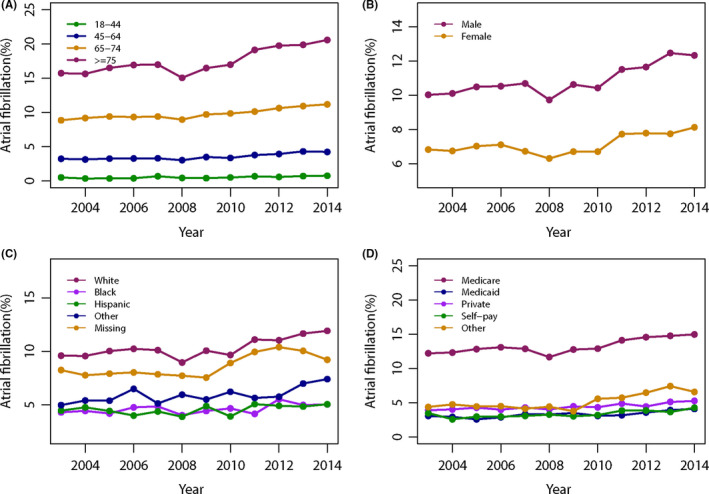
Temporal trends of AF prevalence in metastatic cancer in subgroups categorized by age (A), gender (B), race (C), and income (D)

Overall, the rate of AF in metastatic cancer patients increased significantly from 8.28% in 2003 to 10.06% in 2014 (*p* for trend <0.0001) (Supplemental eTable 4). Figure 1 shows the temporal trend of AF prevalence in subgroups categorized by age, gender, race, and income. In all subgroups, AF prevalence consistently increased from 2003 to 2014 (all *p* for trend <0.05). With regards to the primary tumor site, AF prevalence consistently increased over the study period (*p* for trend <0.05) except endometrium cancer (*p* for trend = 0.9944). Higher prevalence was observed in lung (12.40%), bladder (10.25%), and colorectal cancer (8.56%).

### 3.3 Trends in risk factor profiles and clinical outcomes in metastatic cancer with and without AF

Trends in risk factor profiles in patients with and without AF are shown in Supplemental eFigure 1 in the Supplement. The prevalence of most risk factors including hypertension, diabetes, obesity, renal failure, coronary artery disease, and prior stroke has significantly increased from 2003 to 2014 (*p* for trend <0.0001). Moreover, patients with comorbid AF had higher rates of risk factors over the whole study period. In‐hospital mortality (with AF: 17.44% in 2003 to 12.50% in 2014; without AF: 10.72% in 2003 to 7.75% in 2014; Supplemental eFigure 2) and LOS (with AF: 7.89 days in 2003 to 6.34 days in 2014; without AF: 5.53 days in 2003 to 4.70 days in 2014; Supplemental eFigure 3) decreased significantly regardless of AF status. Cost decreased only in those diagnosed with AF (1,7861$ in 2003 to 1,6418$ in 2014; Supplemental eFigure 4).

### 3.4 Association of AF with patient and hospital characteristics in metastatic cancer

The results suggested that aged ≥75 years (odds ratio [OR], 18.26; 95% confidence interval [CI]: 15.50–21.53), white race (OR, 0.52; 95% CI: 0.50–0.55), male (OR, 0.72; 95% CI: 0.70–0.73), Medicare insurance (OR, 0.87; 95% CI: 0.82–0.93), higher income (OR, 1.11; 95% CI: 1.08–1.15), larger hospital bed size (OR, 1.05; 95% CI: 1.01–1.09), urban teaching hospital (OR, 1.12; 95% CI: 1.08–1.17), and major operating room procedure (OR, 1.15; 95% CI: 1.12–1.19) were risk predictors of AF. Higher ECI score and CHA_2_DS_2_‐VAS_C_ score were also significantly associated with increased AF prevalence. Continued description of the association between individual comorbidities and AF in model 3 is shown in Supplemental eTable 5.

Among primary tumor sites, odds of comorbid AF were higher in lung cancer compared to other cancers (all *p* < 0.0001). Metastasis to lymph nodes and respiratory organs were related to higher odds of AF, whereas metastasis to bone & bone marrow, brain & spinal cord, liver and genital organs were related to lower odds of AF.

### 3.5 Association of AF with clinical outcomes in metastatic cancer

Adjusted analyses showed that both mortality and LOS decreased each year, while cost decreased only in those comorbid with AF (Table 2). In‐hospital mortality was higher in patients with AF than without AF across the whole study period (Supplemental eFigure 2). Patients with AF had 48% higher odds of death than those without AF (OR, 1.48; 95% CI: 1.43–1.54). In addition, AF presence was associated with 18% longer LOS and 19% higher cost.

**TABLE 2:** Association of AF with in‐hospital mortality, LOS, and total cost in metastatic cancer patients

Outcomes	Model a^a^		Model b^b^	
	Estimate	*p*‐value	Estimate	*p*‐value
Association of AF with each outcome among all participants				
Died (OR, 95% CI)	1.61 (1.55, 1.67)	<0.0001	1.48 (1.43, 1.54)	<0.0001
Total cost	0.22	<0.0001	0.19	<0.0001
LOS	0.21	<0.0001	0.18	<0.0001
Association of each outcome with a unit increase in a year in participants with comorbid AF				
Died (OR, 95% CI)	0.94 (0.92, 0.95)	<0.0001	0.94 (0.92,0.96)	<0.0001
Total cost	−0.01	<0.0001	−0.01	<0.0001
LOS	−0.02	<0.0001	−0.03	<0.0001
Association of each outcome with unit increase in year in participants without comorbid AF				
Died (OR, 95% CI)	0.94 (0.92, 0.96)	<0.0001	0.94 (0.91, 0.96)	<0.0001
Total cost	<0.001	0.6300	0.001	0.4400
LOS	−0.02	<0.0001	−0.03	<0.0001

Abbreviations: CI, confidence interval; LOS, length of stay; OR, odds ratio.

^a^ Model a adjusted for age, race, gender, income, insurancetype, year, hospital region, hospital type, hospital bed size, primary tumorsite, multiple metastatic sites (≥2), major operating room procedure, chemotherapy, long‐term anticoagulants, CHA2DS2‐VASC score, metastatic sites and ECI score.

^b^ Model b adjusted for age, race, gender, income, insurance type, year, hospital region, hospital type, hospital bed size, primary tumorsite, multiple metastatic sites (≥2), major operating room procedure, chemotherapy, long‐term anticoagulants, metastatic sites, coronary artery disease, prior stroke, acquired immune deficiency syndrome (AIDS), alcoholabuse, deficiency anemias, rheumatoid arthritis, chronic blood loss anemia, congestive heart failure, chronic pulmonary disease, coagulopathy, depression, uncomplicated diabetes, diabetes with chronic complications, drug abuse, hypertension, hypothyroidism, obesity, lymphoma, fluid and electrolyte disorders, other neurological disorders, paralysis, peripheral vasculardisorders, psychoses, pulmonary circulation disorders, renal failure, ulcerdisease, valvular disease, weight loss and liver disease.

### 3.6 Subgroup and sensitivity analyses

Subgroup analysis based on tumor factors suggested that there were worse outcomes in patients’ comorbid with AF for all types of cancers, with the exception of mortality for prostate tumor site and metastasis to urinary organs (Table 3). Subgroup analyses based on patient characteristics (Supplemental eTable 6) and sensitivity analyses (Supplemental eTable 7) yielded similar results as the primary findings.

**TABLE 3:** Association of AF with in‐hospital mortality, LOS, and total cost in metastatic cancer patients by tumor factors

Tumor factors	Death^a^		LOS		Cost	
	OR (95% CI)	*p*‐value	Estimate	*p*‐value	Estimate	*p*‐value
Primary tumor site						
Lung	1.45 (1.38, 1.52)	<0.0001	0.19	<0.0001	0.21	<0.0001
Stomach	1.54 (1.33, 1.80)	<0.0001	0.21	<0.0001	0.27	<0.0001
Pancreas	1.26 (1.13, 1.42)	<0.0001	0.14	<0.0001	0.15	<0.0001
Colon/rectum	1.48 (1.35, 1.62)	<0.0001	0.13	<0.0001	0.17	<0.0001
Prostate	1.00 (0.82, 1.21)	0.9786	0.13	<0.0001	0.09	0.0002
Bladder	1.30 (1.03, 1.63)	0.0259	0.15	<0.0001	0.16	<0.0001
Breast	1.43 (1.15, 1.78)	0.0012	0.14	<0.0001	0.10	<0.0001
Endometrium	1.62 (1.10, 2.38)	0.0145	0.17	<0.0001	0.13	<0.0001
Ovary	1.84 (1.50, 2.25)	<0.0001	0.20	<0.0001	0.22	<0.0001
Kidney	1.64 (1.32, 2.03)	<0.0001	0.18	<0.0001	0.20	<0.0001
Metastatic sites						
Bone & bone marrow	1.48 (1.39, 1.58)	<0.0001	0.15	<0.0001	0.17	<0.0001
Brain & spinal cord	1.45 (1.32, 1.60)	<0.0001	0.13	<0.0001	0.17	<0.0001
Lymph nodes	1.68 (1.56, 1.82)	<0.0001	0.23	<0.0001	0.24	<0.0001
Liver	1.38 (1.30, 1.47)	<0.0001	0.14	<0.0001	0.17	<0.0001
Respiratory organs	1.53 (1.42, 1.65)	<0.0001	0.17	<0.0001	0.20	<0.0001
Urinary organs	1.26 (0.98, 1.63)	0.0752	0.15	<0.0001	0.15	<0.0001
Adrenal glands	1.47 (1.27, 1.72)	<0.0001	0.19	<0.0001	0.22	<0.0001
Gastrointestinal organs	1.49 (1.34, 1.65)	<0.0001	0.16	<0.0001	0.19	<0.0001
Genital organs	1.65 (1.15, 2.38)	0.0072	0.16	<0.0001	0.20	<0.0001
Other organs	1.62 (1.45, 1.81)	<0.0001	0.22	<0.0001	0.24	<0.0001
Number of metastatic sites						
<2	1.53 (1.46, 1.60)	<0.0001	0.20	<0.0001	0.21	<0.0001
≥2	1.51 (1.42, 1.60)	<0.0001	0.17	<0.0001	0.19	<0.0001

Abbreviations: CI, confidence interval; LOS, length of stay; OR, odds ratio.

^a^ Adjusted for age, race, gender, income, insurance type, year, hospital region, hospital type, hospital bed size, primary tumor site, multiple metastatic sites (≥2), major operating room procedure, chemotherapy, long‐term anticoagulants, metastatic sites, coronary artery disease, prior stroke, acquired immune deficiency syndrome (AIDS), alcohol abuse, deficiency anemias, rheumatoid arthritis, chronic blood loss anemia, congestive heart failure, chronic pulmonary disease, coagulopathy, depression, uncomplicated diabetes, diabetes with chronic complications, drug abuse, hypertension, hypothyroidism, obesity, lymphoma, fluid and electrolyte disorders, other neurological disorders, paralysis, peripheral vascular disorders, psychoses, pulmonary circulation disorders, renal failure, ulcer disease, valvular disease, weight loss and liver disease.

## 4 DISCUSSION

The study using the largest inpatient database in the United States described the temporal trend in AF prevalence, determined characteristics associated with AF, and evaluated the impact of AF on clinical outcomes in hospitalized metastatic cancer patients. Among 2,478,598 patients with 10 most common solid‐organ metastatic malignancies, 8.74% (216,737) were diagnosed with AF. We also observed a significant increasing trend in AF prevalence. Patient‐, hospital‐, and tumor‐related factors could independently predict AF occurrence. Finally, AF in metastatic cancer was related to worse prognosis and higher healthcare resource utilization.

Leading this discussion are the rapidly increasing prevalence of the two public health problems: AF and cancer. The interconnections between AF and cancer are complicated with poorly understood mechanisms.^8^, ^9^, ^10^ On the one hand, AF was frequently observed in patients with cancer, especially in those undergoing pulmonary resection for lung cancer (ranged from 5.6% to 28%).^9^, ^11^ Some drugs for cancer could induce AF, which covered cytotoxic agents, high‐dose corticosteroids, antiemetic agents, and targeted therapies.^19^ Additionally, outside the postoperative period or prior to any treatment, cancer itself may be regarded as a comorbid state that predisposed to AF occurrence as cancer patients shared several risk factors with AF including advancing age, hypoxia, and common comorbidities such as smoking, obesity, and hypertension.^20^, ^21^ Autonomic nervous system imbalance, paraneoplastic conditions as well as direct invasiveness of cancer may also explain the higher AF prevalence in cancer patients.^10^, ^22^ Both conditions may share common characteristics of chronic inflammation.^23^ On the other hand, AF appeared to be a risk marker of occult cancer. Conen et al. conducted a large, long‐term prospective cohort study and concluded that women with new‐onset AF had a significantly increased cancer risk after adjusting for potential confounders.^24^ Overall, the underlying pathogenetic mechanisms with regards to the association between AF and cancer need to be further elucidated.

Increasing AF prevalence in hospitalized metastatic cancer may be attributed to multiple factors. At first glance, the aging population in the United States may play a role in the increased incidence and prevalence of AF, especially in patients with metastatic cancer.^25^ Our analysis showed that more than half of AF patients aged ≥75 years old. On another note, improved survival (decreasing in‐hospital mortality) in metastatic cancer patients made recurrent hospital admissions possible, which indirectly resulted in an increasing prevalence of AF hospital encounters.^26^ Advanced detection technology and increased surveillance for AF may also be involved through the improved detection rate.^2^ Concurrently, the prevalence of most coexisting comorbidities in metastatic cancer patients has significantly increased, such as coronary artery disease, congestive heart failure, obesity, diabetes, and hypertension.^2^, ^25^ These risk factors are more frequently observed in cancer patients and may thus be linked to increased AF prevalence.

Several factors significantly predict AF in hospitalized metastatic cancer patients. Older age, white race, male, Medicare insurance, higher income, and admission to an urban teaching hospital or hospital in the Northeast were related to higher AF prevalence, consistent with previous publications focusing on other conditions.^27^ Higher ECI or the presence of conditions like coronary artery disease, prior stroke, hypertension, obesity or congestive heart failure may reflect higher severity of comorbidities burdens and therefore were associated with increased odds of AF. The results also suggested that cancer patients with major operating room procedure were more likely to be diagnosed with AF, which supported the evidence of higher post‐operative prevalence of AF in cancer patients in countless epidemiological studies.^9^, ^10^ Considering primary tumor sites, lung cancer had the highest AF prevalence (12.40%), which is consistent with previous evidence, especially the higher AF rate in lung cancer patients undergoing pulmonary resection.^9^, ^10^ Furthermore, metastasis to lymph nodes and respiratory organs was related to higher odds of AF, whereas metastasis to bone & bone marrow, brain & spinal cord, liver and genital organs was related to lower odds of AF. In the light of our present knowledge, this study for the first time examined the independent association between specific invasion sites of cancer and AF occurrence. Possible underlying mechanisms need to be assessed in further studies.

The decreasing trends of in‐hospital mortality and LOS in metastatic cancer patients both with and without AF are encouraging. Particularly, declines in patients with AF were more pronounced. The results also suggested that AF presence was a risk predictor for in‐hospital mortality. However, previous publications focusing on the prognosis of AF in cancer patients are conflicting. Imperatori et al. reported an increased post‐operative mortality after lung cancer surgery, while Walsh et al. found that AF was not an independent predictor for mortality in patients with colorectal cancer.^28^, ^29^ Small sample size or different types and severity of cancers in previous analyses may account for the inconsistent results. The present study with a larger relatively homogeneous sample focused on metastatic cancer patients. Our subgroup analysis by cancer types indicated that AF was a risk predictor for all types of cancers except for prostate cancer.

The current study has several strengthens which allowed for a comprehensive evaluation of temporal trends, characteristics, and outcomes of metastatic cancer patients comorbid with AF. There are also limitations. First, the identification of cancer and metastatic sites was based on ICD‐9‐CM codes. There was a possibility of undercoding or miscoding in the administrative database. Metastatic status may be accurately ascertained through the review of medical records or other confirmatory data.^30^ Second, we could not distinguish index admissions from readmitted hospitalizations because a unique patient identifier was not available in the NIS. In addition, readmission rates between metastatic cancer with and without AF were unknown so our analysis may underestimate or overestimate the true AF prevalence among individual metastatic cancer patients. Third, unmeasured confounders such as medications that could be intrinsically related to both AF and outcomes may potentially contribute to some of the observable differences. Nevertheless, the results were proved robust and reliable in subgroup and sensitivity analyses from different statistical models.

## 5 CONCLUSIONS

Patients with AF represent 8.74% of patients diagnosed with metastatic cancer. AF prevalence continued to increase from 2003 to 2014. Accounting for potential confounders, AF is linked to poorer prognosis and higher healthcare resource utilization. As the population ages, high‐quality preventive and treatment management strategies are needed for metastatic cancer comorbid with AF.

## CONFLICTS OF INTEREST

The authors made no disclosures.

## AUTHOR CONTRIBUTIONS

Hedong Han: conceptualization, formal analysis, writing‐original draft. Longpei Chen: conceptualization, writing‐original draft. Zhen Lin: conceptualization, formal analysis. Xin Wei: conceptualization, writing‐original draft. Wei Guo: conceptualization and writing‐review and editing. Yamei Yu: conceptualization and writing‐review and editing. Cheng Wu: conceptualization and writing‐review and editing. Yang Cao: conceptualization and writing‐review and editing. Jia He: conceptualization, data curation, supervision, and writing‐review and editing.

## ETHICAL APPROVAL

The NIS is a publicly available, de‐identified database that does not contain personal information; thus, requirements for the Institutional Review Board approval and informed consent are waived by the Second Military Medical University.

## Supporting information

Supplementary Material

## Data Availability

The datasets used and analyzed are available from the HCUP website: https://www.hcup‐us.ahrq.gov/databases.jsp.
