# Erratum

**DOI:** 10.1002/cam4.4305

**Published:** 2021-10-01

In the article by Han et al.,^1^ the authors would like to update the order of the author affiliations.

Hedong Han^1,2^, Longpei Chen^3^, Zhen Lin^1^, Xin Wei^4,5^, Wei Guo^1^, Yamei Yu^6^, Cheng Wu^1^, Yang Cao^7^, Jia He^1,8^

^1^Department of Health Statistics, Second Military Medical University, Shanghai 200433, China.

^2^Department of Respiratory and Critical Care Medicine, Jinling Hospital, Nanjing University School of Medicine, Nanjing 210002, China.

^3^Department of Medical Oncology, Shanghai Changhai Hospital, Second Military Medical University, Shanghai 200433, China.

^4^Department of Cardiology, Virginia Commonwealth University, 1250 E Marshall Street, Richmond, Virginia, 23298, USA.

^5^Mount Sinai St. Luke’s and West Medical Center, 1111 Amsterdam Ave. New York NY 10025, USA.

^6^Department of Cardiology, Shanghai Changning District Central Hospital, Shanghai 200433, China.

^7^Clinical Epidemiology and Biostatistics, School of Medical Sciences, Örebro University, 701 82 Örebro, Sweden.

^8^Tongji University School of Medicine, Shanghai 200092, China.

REFERENCE

1. Han H, Chen L, Lin Z, et al. Prevalence, trends, and outcomes of atrial fibrillation in hospitalized patients with metastatic cancer: findings from a national sample. Cancer Med. 2021;10:5661–5670. https://doi.org/10.1002/cam4.4105

