# Supplementary material for: Prevalence, trends, and outcomes of atrial fibrillation in hospitalized patients with metastatic cancer: findings from a national sample
Source: Cancer Med. 2021 Jul 7;10(16):5661–70. doi: 10.1002/cam4.4105 (PMC8366074; doi:10.1002/cam4.4105)
Supplement: Supplementary file 1 — Supplementary Material [file CAM4-10-5661-s001.docx]

**eFigure Legends**

eFigure 1. Trends in risk factor profiles in metastatic cancer with and without AF. (A. hypertension; B. diabetes; C. congestive heart failure; D. obesity; E. renal failure; F. coronary artery disease; G. prior stroke; H. valvular disease; I. long-term anticoagulants.)

eFigure 2. Trends in in-hospital mortality in metastatic cancer with and without AF

eFigure 3. Trends in length of stay in metastatic cancer with and without AF

eFigure 4. Trends in hospitalization cost in metastatic cancer with and without AF

**eTable Legends**

eTable 1. International Classification of Diseases, Ninth Revision, Clinical Modification (ICD-9-CM) codes used to identify metastatic cancer

eTable 2. Summary of missing data in the study

eTable 3. Distribution of Elixhauser comorbid conditions in metastatic cancer patients with and without AF

eTable 4. Weighted prevalence of AF in metastatic cancer patients hospitalized from 2003 to 2014 according to primary tumor site

eTable 5. Risk predictors of comorbid AF in hospitalized metastatic cancer patients

eTable 6. Association of AF with in-hospital mortality, LOS and total cost in metastatic cancer patients based on subgroup analysis

eTable 7. Sensitivity analysis on association of AF with in-hospital mortality, LOS and total cost in metastatic cancer patients


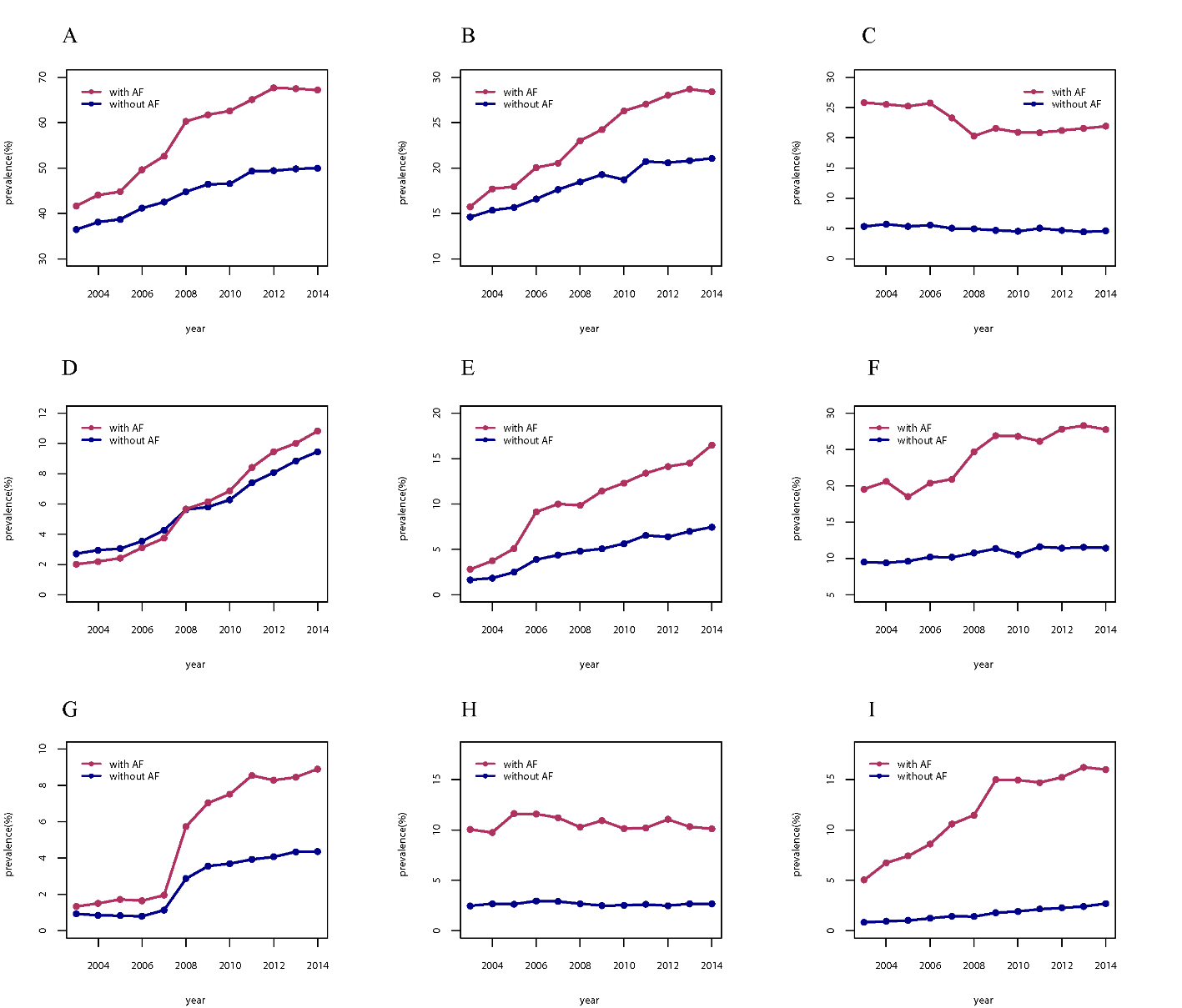


eFigure 1. Trends in risk factor profiles in metastatic cancer with and without AF. (A. hypertension; B. diabetes; C. congestive heart failure; D. obesity; E. renal failure; F. coronary artery disease; G. prior stroke; H. valvular disease; I. long-term anticoagulants.)


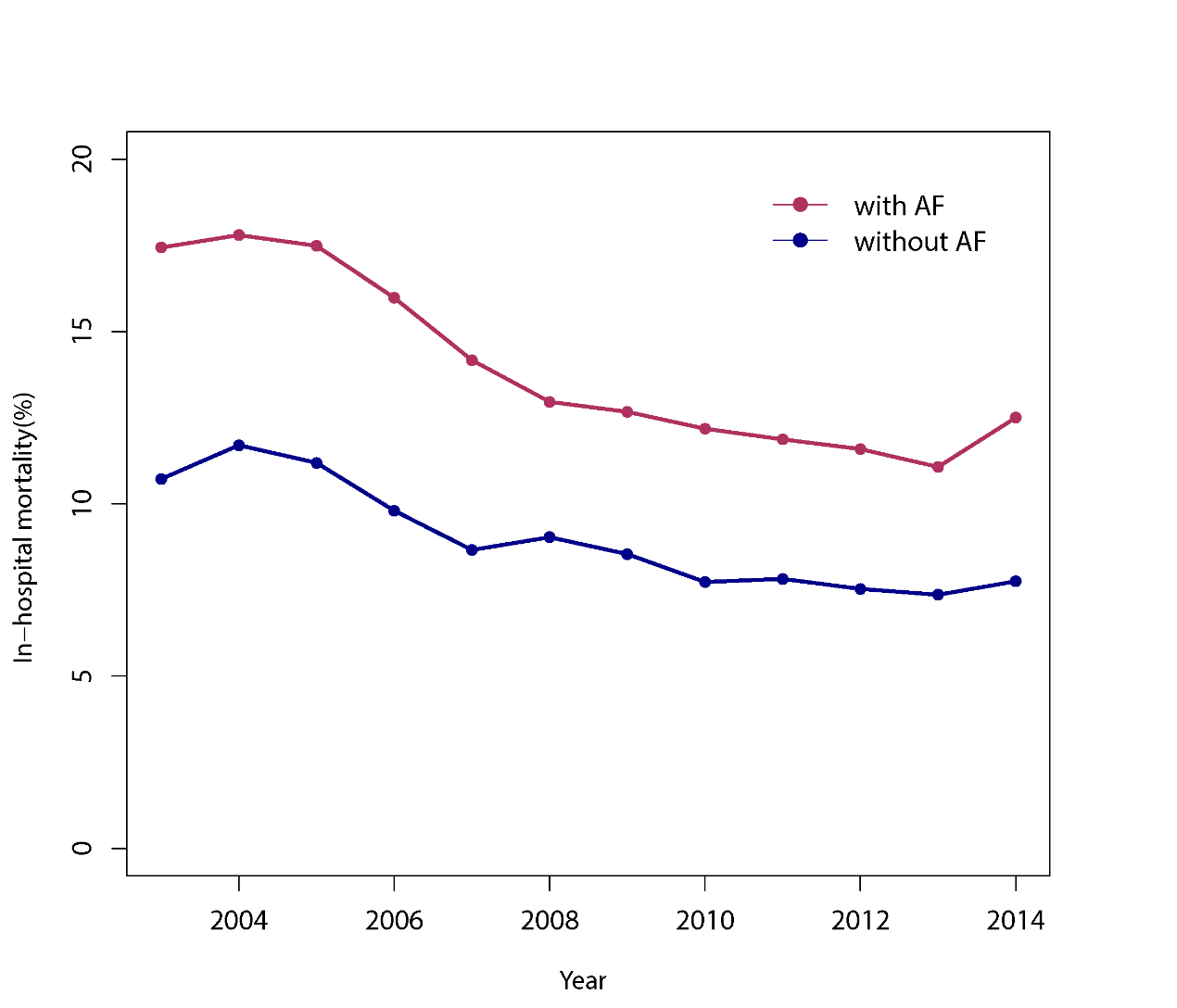


eFigure 2. Trends in in-hospital mortality in metastatic cancer with and without AF


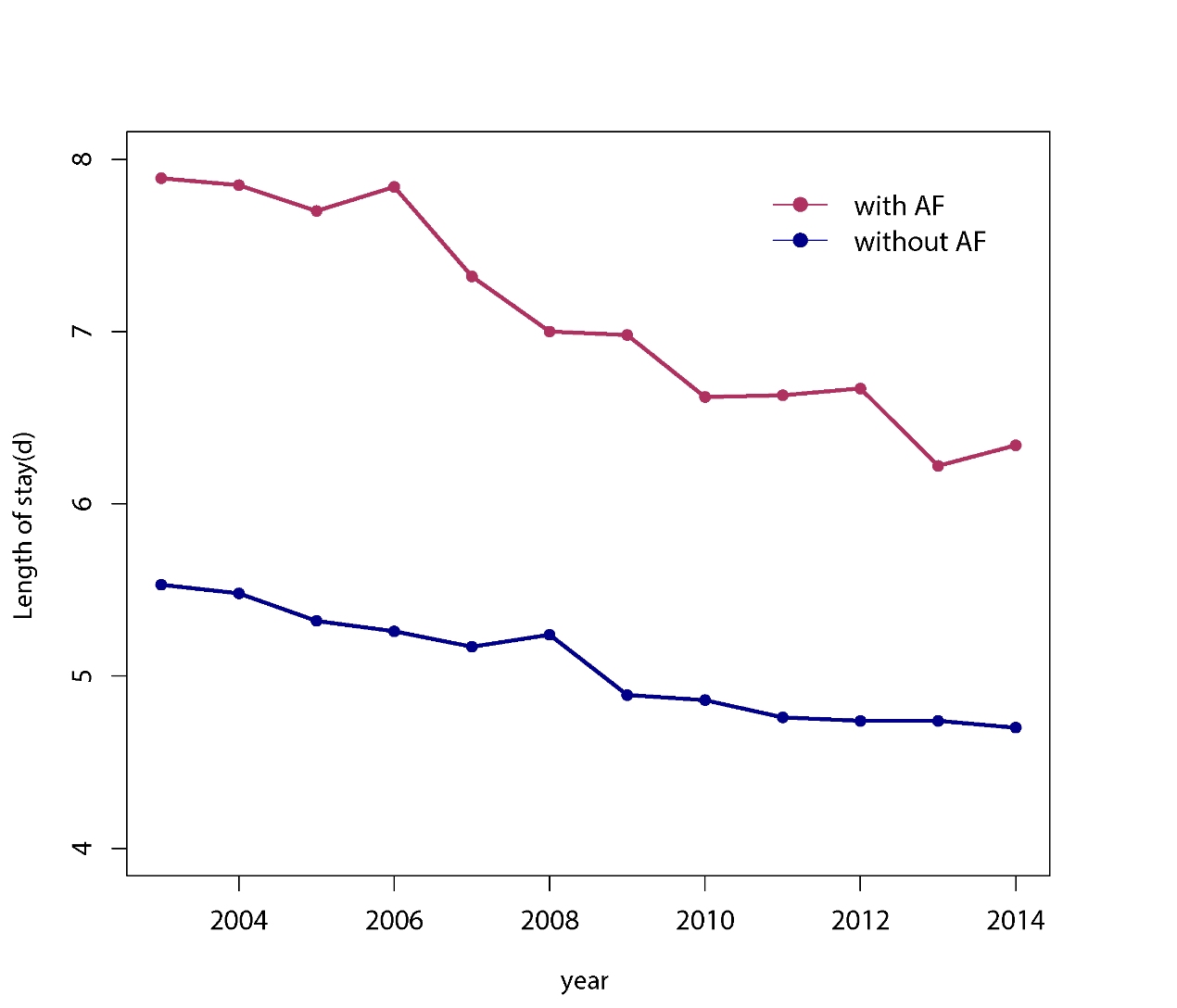


eFigure 3. Trends in length of stay in metastatic cancer with and without AF


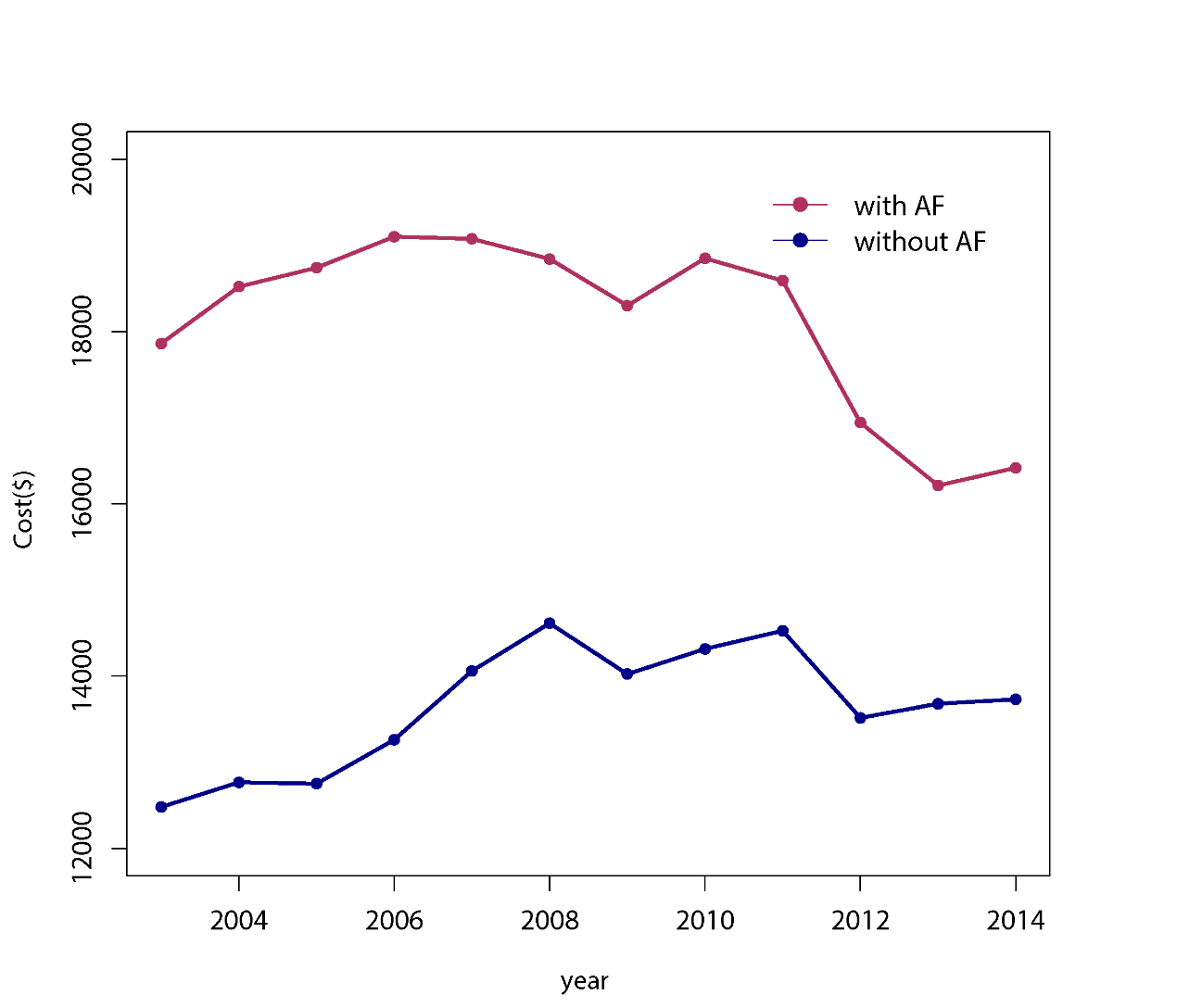


eFigure 4. Trends in hospitalization cost in metastatic cancer with and without AF

**eTable 1. International Classification of Diseases, Ninth Revision, Clinical Modification (ICD-9-CM) codes used to identify** **metastatic cancer**

| **Disease Entity** | **ICD-9-CM Codes** |
| --- | --- |
| **Primary tumor site** |  |
| Lung & bronchus | 162.x |
| Stomach | 151.x |
| Pancreas | 157.x |
| Colon/rectum | 153.x, 154.x |
| Prostate | 185.x |
| Bladder | 188.x |
| Breast | 174.x |
| Endometrium | 182.x |
| Ovary | 183.x |
| Kidney | 189, 189.0, 189.8, 189.9 |
| **Secondary tumor site** |  |
| Bone & bone marrow | 198.5 |
| Brain & spinal cord | 198.3, 198.4 |
| Lymph nodes | 196.x |
| Liver | 197.7 |
| Respiratory organs | 197.0, 197.1, 197.2, 197.3, 197.8 |
| Urinary organs | 198.0, 198.1 |
| Adrenal glands | 198.7 |
| Gastrointestinal organs | 197.4, 197.5, 197.6 |
| Genital organs | 198.82 |
| Other organs | 198.81, 198.89, 198.2 |

**eTable 2. Summary of missing data in the study**

| **Variable** | **Missing data (%)** |
| --- | --- |
| Female | 0.05 |
| Race | 17.13 |
| Payer | 0.20 |
| Income | 2.25 |
| Hospital Bed size | 0.39 |
| Hospital type | 0.39 |
| Elixhauser Comorbidity Index | 0.51 |
| Total cost | 6.45 |
| Death | 0.07 |

**eTable 3. Distribution of Elixhauser comorbid conditions in metastatic cancer patients with and without AF**

| **Variables** | **AF**  **(N=215,434, %)** | **Without AF**  **(N=2,250,700, %)** | P-value |
| --- | --- | --- | --- |
| Acquired immune deficiency syndrome | 141 (0.07) | 4493 (0.20) | <0.0001 |
| Alcohol abuse | 4627 (2.15) | 53854 (2.39) | 0.0021 |
| Deficiency anemias | 52079 (24.17) | 494809 (21.98) | <0.0001 |
| Rheumatoid arthritis/collagen vascular  diseases | 4348 (2.02) | 34078 (1.51) | <0.0001 |
| Chronic blood loss anemia | 8748 (4.06) | 69638 (3.09) | <0.0001 |
| Congestive heart failure | 49315 (22.89) | 113327 (5.04) | <0.0001 |
| Chronic pulmonary disease | 75685 (35.13) | 485558 (21.57) | <0.0001 |
| Coagulopathy | 13712 (6.36) | 96455 (4.29) | <0.0001 |
| Depression | 14966 (6.95) | 165119 (7.34) | 0.0049 |
| Diabetes, uncomplicated | 43909 (20.38) | 368252 (16.36) | <0.0001 |
| Diabetes with chronic complications | 5717 (2.65) | 39896 (1.77) | <0.0001 |
| Drug abuse | 879 (0.41) | 22591 (1.00) | <0.0001 |
| Hypertension, uncomplicated and  complicated | 121984 (56.62) | 989034 (43.94) | <0.0001 |
| Hypothyroidism | 22496 (10.44) | 175107 (7.78) | <0.0001 |
| Liver disease | 4234 (1.97) | 47467 (2.11) | 0.0491 |
| Lymphoma | 1298 (0.60) | 7692 (0.34) | <0.0001 |
| Fluid and electrolyte disorders | 73942 (34.32) | 580049 (25.77) | <0.0001 |
| Other neurological disorders | 11136 (5.17) | 103783 (4.61) | <0.0001 |
| Obesity | 12432 (5.77) | 122054 (5.42) | 0.0047 |
| Paralysis | 3391 (1.57) | 29796 (1.32) | <0.0001 |
| Peripheral vascular disorders | 15688 (7.28) | 78145 (3.47) | <0.0001 |
| Psychoses | 4488 (2.08) | 55280 (2.46) | <0.0001 |
| Pulmonary circulation disorders | 12068 (5.60) | 50043 (2.22) | <0.0001 |
| Renal failure | 21688 (10.07) | 102724 (4.56) | <0.0001 |
| Peptic ulcer disease excluding bleeding | 128 (0.06) | 2077 (0.09) | 0.0279 |
| Valvular disease | 22867 (10.61) | 59796 (2.66) | <0.0001 |
| Weight loss | 28539 (13.25) | 243662 (10.83) | <0.0001 |

Note: 12,464 patients with missing individual conditions were excluded.

**eTable 4. Weighted prevalence of AF in metastatic cancer patients hospitalized from 2003 to 2014 according to primary tumor site**

| Variables | Total | 2003 | 2004 | 2005 | 2006 | 2007 | 2008 | 2009 | 2010 | 2011 | 2012 | 2013 | 2014 | *P* for Trend |
| --- | --- | --- | --- | --- | --- | --- | --- | --- | --- | --- | --- | --- | --- | --- |
| **Total AF number** | 216737 | 19261 | 19046 | 20354 | 18719 | 18865 | 17277 | 17085 | 16835 | 18371 | 17125 | 17030 | 16770 |  |
| **Total % with AF** | 8.74 | 8.28 | 8.24 | 8.56 | 8.65 | 8.48 | 7.83 | 8.47 | 8.35 | 9.41 | 9.52 | 9.89 | 10.06 | <0.0001 |
| **Primary tumor site** |  |  |  |  |  |  |  |  |  |  |  |  |  |  |
| **Lung** | 12.40 | 11.42 | 11.55 | 11.85 | 11.59 | 12.14 | 11.31 | 12.08 | 12.26 | 13.60 | 13.88 | 14.40 | 14.24 | <0.0001 |
| **Stomach** | 8.48 | 9.67 | 7.55 | 8.63 | 8.40 | 7.71 | 8.29 | 8.19 | 7.41 | 8.16 | 8.68 | 9.34 | 10.07 | 0.0331 |
| **Pancreas** | 8.18 | 7.47 | 6.67 | 7.06 | 7.79 | 7.88 | 6.48 | 7.79 | 8.69 | 9.55 | 9.27 | 9.40 | 10.18 | <0.0001 |
| **Colon/rectum** | 8.56 | 8.43 | 8.52 | 9.01 | 8.79 | 8.62 | 7.63 | 8.39 | 7.70 | 9.02 | 8.76 | 9.07 | 8.96 | 0.0354 |
| **Prostate** | 8.15 | 6.96 | 7.72 | 7.71 | 8.74 | 8.71 | 7.83 | 7.92 | 7.39 | 8.37 | 8.88 | 9.38 | 9.36 | <0.0001 |
| **Bladder** | 10.25 | 8.50 | 9.92 | 8.99 | 10.22 | 10.33 | 7.93 | 9.79 | 11.26 | 11.25 | 10.59 | 12.08 | 13.05 | <0.0001 |
| **Breast** | 3.52 | 2.99 | 3.15 | 3.44 | 3.30 | 3.17 | 3.62 | 3.18 | 3.65 | 3.81 | 4.12 | 4.14 | 4.46 | <0.0001 |
| **Endometrium** | 5.58 | 7.20 | 5.23 | 5.56 | 5.57 | 3.95 | 4.94 | 5.61 | 5.66 | 5.93 | 6.62 | 5.94 | 5.20 | 0.9944 |
| **Ovary** | 5.17 | 4.92 | 5.02 | 5.33 | 5.77 | 4.48 | 4.57 | 5.06 | 4.51 | 5.40 | 5.46 | 5.95 | 5.84 | 0.0003 |
| **Kidney** | 7.46 | 6.59 | 7.19 | 6.99 | 8.73 | 7.02 | 5.39 | 7.35 | 6.70 | 7.58 | 8.91 | 8.49 | 8.94 | <0.0001 |

**eTable 5. Risk predictors of comorbid AF in hospitalized metastatic cancer patients**

| Variables | Unadjusted model 1 | | Multivariable model 2**^#^** | | Multivariable model 3* | |
| --- | --- | --- | --- | --- | --- | --- |
|  | OR (95 % CI) | *P*-value | OR (95 % CI) | *P*-value | OR (95 % CI) | *P*-value |
| **Year, per unit increase** | 1.02(1.02,1.02) | <0.0001 | 1.00(1.00,1.00) | 0.7900 | 1.01(1.00,1.01) | <0.0001 |
| **Age, years** |  |  |  |  |  |  |
| Per unit increase | 1.07(1.07,1.07) | <0.0001 | 1.05(1.05,1.05) | <0.0001 | 1.06(1.06,1.06) | <0.0001 |
| 45-64 vs 18-44 | 6.88(5.85,8.10) | <0.0001 | 4.52(3.83,5.33) | <0.0001 | 4.83(4.10,5.68) | <0.0001 |
| 65-74 vs 18-44 | 20.45(17.39,24.05) | <0.0001 | 8.60(7.28,10.17) | <0.0001 | 10.36(8.79,12.22) | <0.0001 |
| ≥75 vs 18-44 | 39.42(33.50,46.37) | <0.0001 | 13.73(11.58,16.27) | <0.0001 | 18.26(15.50,21.53) | <0.0001 |
| **Race** |  |  |  |  |  |  |
| Black vs white | 0.42(0.40,0.44) | <0.0001 | 0.50(0.47,0.52) | <0.0001 | 0.52(0.50,0.55) | <0.0001 |
| Hispanic vs white | 0.42(0.38,0.45) | <0.0001 | 0.55(0.52,0.59) | <0.0001 | 0.59(0.55,0.63) | <0.0001 |
| Others vs white | 0.55(0.51,0.58) | <0.0001 | 0.66(0.62,0.70) | <0.0001 | 0.69(0.65,0.74) | <0.0001 |
| Missing vs white | 0.77(0.74,0.81) | <0.0001 | 0.90(0.87,0.94) | <0.0001 | 0.91(0.88,0.95) | <0.0001 |
| **Insurance type** |  |  |  |  |  |  |
| Medicaid vs Medicare | 0.22(0.21,0.24) | <0.0001 | 0.85(0.80,0.91) | <0.0001 | 0.87(0.82,0.93) | <0.0001 |
| Private vs Medicare | 0.30(0.29,0.31) | <0.0001 | 0.90(0.87,0.93) | <0.0001 | 0.89(0.86,0.93) | <0.0001 |
| Self-pay vs Medicare | 0.23(0.21,0.25) | <0.0001 | 0.80(0.73,0.87) | <0.0001 | 0.80(0.73,0.88) | <0.0001 |
| Others vs Medicare | 0.36(0.32,0.39) | <0.0001 | 0.86(0.79,0.94) | 0.0011 | 0.87(0.79,0.95) | 0.0028 |
| **Female** | 0.63(0.62,0.64) | <0.0001 | 0.60(0.59,0.62) | <0.0001 | 0.72(0.70,0.73) | <0.0001 |
| **Income quartile** |  |  |  |  |  |  |
| Q2 vs Q1 | 1.17(1.14,1.21) | <0.0001 | 1.06(1.03,1.09) | 0.0003 | 1.06(1.03,1.10) | 0.0001 |
| Q3 vs Q1 | 1.20(1.16,1.24) | <0.0001 | 1.08(1.04,1.11) | <0.0001 | 1.08(1.04,1.12) | <0.0001 |
| Q4 vs Q1 | 1.23(1.18,1.28) | <0.0001 | 1.12(1.08,1.15) | <0.0001 | 1.11(1.08,1.15) | <0.0001 |
| **Hospital bed size** |  |  |  |  |  |  |
| Medium vs small | 1.04(0.98,1.09) | 0.2226 | 1.03(0.98,1.07) | 0.2868 | 1.02(0.97,1.07) | 0.4834 |
| Large vs small | 1.00(0.95,1.05) | 0.8607 | 1.05(1.01,1.10) | 0.0117 | 1.05(1.01,1.09) | 0.0245 |
| **Hospital type** |  |  |  |  |  |  |
| Urban nonteaching vs rural | 1.07(1.02,1.11) | 0.0034 | 1.07(1.03,1.12) | 0.0014 | 1.07(1.03,1.12) | 0.0010 |
| Urban teaching vs rural | 0.92(0.88,0.97) | 0.0004 | 1.11(1.06,1.16) | <0.0001 | 1.12(1.08,1.17) | <0.0001 |
| **Hospital region** |  |  |  |  |  |  |
| Midwest vs northeast | 1.02(0.97,1.07) | 0.4900 | 0.97(0.93,1.01) | 0.1176 | 0.96(0.93,1.00) | 0.0468 |
| South vs northeast | 0.89(0.84,0.93) | <0.0001 | 0.95(0.92,0.98) | 0.0032 | 0.95(0.92,0.98) | 0.0043 |
| West vs northeast | 0.88(0.83,0.93) | <0.0001 | 0.94(0.90,0.97) | 0.0011 | 0.95(0.91,0.99) | 0.0140 |
| **Primary tumor site** |  |  |  |  |  |  |
| Stomach vs Lung | 0.65(0.62,0.69) | <0.0001 | 0.64(0.61,0.68) | <0.0001 | 0.68(0.65,0.72) | <0.0001 |
| Pancreas vs Lung | 0.63(0.60,0.66) | <0.0001 | 0.54(0.52,0.57) | <0.0001 | 0.60(0.58,0.63) | <0.0001 |
| Colon/rectum vs Lung | 0.66(0.64,0.68) | 0.0034 | 0.59(0.57,0.62) | <0.0001 | 0.61(0.59,0.63) | <0.0001 |
| Prostate vs Lung | 0.63(0.59,0.67) | <0.0001 | 0.47(0.44,0.51) | <0.0001 | 0.48(0.45,0.52) | <0.0001 |
| Bladder vs Lung | 0.81(0.76,0.86) | <0.0001 | 0.53(0.49,0.56) | <0.0001 | 0.53(0.50,0.57) | <0.0001 |
| Breast vs Lung | 0.26(0.24,0.27) | <0.0001 | 0.40(0.38,0.43) | <0.0001 | 0.42(0.40,0.44) | <0.0001 |
| Endometrium vs Lung | 0.42(0.39,0.45) | <0.0001 | 0.51(0.47,0.55) | <0.0001 | 0.54(0.49,0.59) | <0.0001 |
| Ovary vs Lung | 0.39(0.36,0.41) | <0.0001 | 0.50(0.47,0.54) | <0.0001 | 0.52(0.48,0.56) | <0.0001 |
| Kidney vs Lung | 0.57(0.54,0.61) | <0.0001 | 0.56(0.52,0.59) | <0.0001 | 0.57(0.53,0.61) | <0.0001 |
| **Multiple metastatic sites** | 0.89(0.87,0.91) | <0.0001 | 1.00(0.96,1.05) | 0.9327 | 1.01(0.96,1.05) | 0.8425 |
| **Major operating room procedure** | 0.89(0.87,0.91) | <0.0001 | 1.13(1.10,1.16) | <0.0001 | 1.15(1.12,1.19) | <0.0001 |
| **Chemotherapy** | 0.91(0.86,0.96) | 0.0005 | 1.00(0.95,1.06) | 0.8991 | 0.98(0.93,1.04) | 0.5532 |
| **Long-term anticoagulants** | 8.04(7.68,8.41) | <0.0001 | 7.00(6.69,7.33) | <0.0001 | 6.77(6.45,7.09) | <0.0001 |
| **CHA_2_DS_2_-VASc score** | 1.52(1.50,1.53) | <0.0001 | 1.16(1.15,1.18) | <0.0001 |  |  |
| **Metastatic site** |  |  |  |  |  |  |
| Bone & bone marrow | 1.04(1.01,1.07) | 0.0048 | 0.88(0.85,0.92) | <0.0001 | 0.89(0.85,0.92) | <0.0001 |
| Brain & spinal cord | 0.79(0.76,0.82) | <0.0001 | 0.68(0.65,0.72) | <0.0001 | 0.73(0.70,0.77) | <0.0001 |
| Lymph nodes | 0.96(0.94,0.98) | 0.0002 | 1.09(1.05,1.13) | <0.0001 | 1.10(1.05,1.14) | <0.0001 |
| Liver | 0.92(0.89,0.94) | <0.0001 | 0.89(0.86,0.93) | <0.0001 | 0.89(0.86,0.93) | <0.0001 |
| Respiratory organs | 1.21(1.17,1.24) | <0.0001 | 1.10(1.06,1.14) | <0.0001 | 1.09(1.05,1.13) | <0.0001 |
| Urinary organs | 0.93(0.87,0.99) | 0.0188 | 0.99(0.92,1.07) | 0.8659 | 1.00(0.93,1.08) | 0.9454 |
| Adrenal glands | 1.12(1.07,1.18) | <0.0001 | 0.91(0.86,0.97) | 0.0032 | 0.93(0.87,0.99) | 0.0193 |
| Gastrointestinal organs  (other than liver) | 0.74(0.72,0.77) | <0.0001 | 0.97(0.93,1.01) | 0.1717 | 0.97(0.92,1.01) | 0.1014 |
| Genital organs | 0.57(0.53,0.61) | <0.0001 | 0.87(0.80,0.93) | 0.0002 | 0.86(0.80,0.93) | 0.0001 |
| Other organs | 1.01(0.98,1.05) | 0.4478 | 1.11(1.06,1.16) | <0.0001 | 1.11(1.06,1.16) | <0.0001 |
| **ECI score** |  |  |  |  |  |  |
| 1 vs 0 | 2.24(2.12,2.35) | <0.0001 | 1.54(1.46,1.62) | <0.0001 |  |  |
| 2 vs 0 | 3.24(3.08,3.42) | <0.0001 | 1.83(1.74,1.93) | <0.0001 |  |  |
| ≥3 vs 0 | 5.24(4.97,5.52) | <0.0001 | 2.42(2.29,2.56) | <0.0001 |  |  |
| **Coronary artery disease** | 2.66(2.58,2.73) | <0.0001 |  |  | 1.23(1.20,1.27) | <0.0001 |
| **Prior stroke** | 2.11(2.01,2.22) | <0.0001 |  |  | 1.24(1.17,1.31) | <0.0001 |
| **AIDS** | 0.33(0.23,0.48) | <0.0001 |  |  | 0.87(0.59,1.27) | 0.4590 |
| **Alcohol abuse** | 0.90(0.83,0.96) | 0.0021 |  |  | 1.04(0.97,1.12) | 0.2972 |
| **Deficiency anemias** | 1.13(1.10,1.16) | <0.0001 |  |  | 0.96(0.93,0.98) | 0.0012 |
| **Rheumatoid arthritis** | 1.34(1.24,1.45) | <0.0001 |  |  | 1.08(0.99,1.17) | 0.0727 |
| **Chronic blood loss anemia** | 1.33(1.26,1.40) | <0.0001 |  |  | 1.09(1.03,1.15) | 0.0038 |
| **Congestive heart failure** | 5.60(5.44,5.77) | <0.0001 |  |  | 3.13(3.04,3.24) | <0.0001 |
| **Chronic pulmonary disease** | 1.97(1.92,2.02) | <0.0001 |  |  | 1.24(1.21,1.27) | <0.0001 |
| **Coagulopathy** | 1.52(1.46,1.59) | <0.0001 |  |  | 1.37(1.30,1.43) | <0.0001 |
| **Depression** | 0.94(0.91,0.98) | 0.0047 |  |  | 0.94(0.90,0.98) | 0.0021 |
| **Diabetes, uncomplicated** | 1.31(1.28,1.34) | <0.0001 |  |  | 1.03(1.00,1.06) | 0.0487 |
| **Diabetes with chronic complications** | 1.51(1.41,1.62) | <0.0001 |  |  | 0.97(0.90,1.05) | 0.4079 |
| **Drug abuse** | 0.40(0.35,0.47) | <0.0001 |  |  | 0.80(0.69,0.94) | 0.0072 |
| **Hypertension** | 1.67(1.63,1.71) | <0.0001 |  |  | 1.11(1.08,1.13) | <0.0001 |
| **Hypothyroidism** | 1.38(1.34,1.43) | <0.0001 |  |  | 1.08(1.04,1.12) | <0.0001 |
| **Obesity** | 1.07(1.02,1.12) | 0.0048 |  |  | 1.19(1.14,1.25) | <0.0001 |
| **Lymphoma** | 1.77(1.55,2.02) | <0.0001 |  |  | 1.23(1.06,1.42) | 0.0060 |
| **Fluid and electrolyte disorders** | 1.51(1.47,1.54) | <0.0001 |  |  | 1.30(1.26,1.33) | <0.0001 |
| **Other neurological disorders** | 1.13(1.08,1.18) | <0.0001 |  |  | 0.95(0.91,1.00) | 0.0557 |
| **Paralysis** | 1.19(1.10,1.30) | <0.0001 |  |  | 1.01(0.92,1.11) | 0.8391 |
| **Peripheral vascular disorders** | 2.18(2.09,2.28) | <0.0001 |  |  | 1.04(0.99,1.09) | 0.0992 |
| **Psychoses** | 0.85(0.79,0.91) | <0.0001 |  |  | 0.91(0.84,0.98) | 0.0097 |
| **Pulmonary circulation Disorders** | 2.61(2.49,2.74) | <0.0001 |  |  | 1.49(1.41,1.57) | <0.0001 |
| **Renal failure** | 2.34(2.26,2.43) | <0.0001 |  |  | 1.18(1.13,1.23) | <0.0001 |
| **Ulcer disease** | 0.65(0.43,0.96) | 0.0301 |  |  | 0.53(0.35,0.81) | 0.0033 |
| **Valvular disease** | 4.35(4.18,4.53) | <0.0001 |  |  | 2.17(2.08,2.27) | <0.0001 |
| **Weight loss** | 1.26(1.22,1.30) | <0.0001 |  |  | 1.07(1.03,1.11) | 0.0005 |
| **Liver disease** | 0.93(0.87,1.00) | 0.0492 |  |  | 1.04(0.96,1.13) | 0.3109 |

Abbreviations: OR, odds ratio; CI, confidence interval; Q1, 0-25^th^ Percentile, Q2, 20-50^th^ Percentile, Q3, 50-75^th^ Percentile, Q4, 75-100^th^ Percentile; ECI, Elixhauser comorbidity index.

^#^ Model 2 adjusted for age, race, gender, income, insurance type, year, hospital region, hospital type, hospital bed size, primary tumor site, multiple metastatic sites (≥2), major operating room procedure, chemotherapy, long-term anticoagulants, CHA2DS2-VASC score, metastatic site and ECI score.

* Model 3 adjusted for age, race, gender, income, insurance type, year, hospital region, hospital type, hospital bed size, primary tumor site, multiple metastatic sites (≥2), major operating room procedure, chemotherapy, long-term anticoagulants, metastatic site, coronary artery disease, prior stroke, acquired immune deficiency syndrome (AIDS), alcohol abuse, deficiency anemias, rheumatoid arthritis, chronic blood loss anemia, congestive heart failure, chronic pulmonary disease, coagulopathy, depression, uncomplicated diabetes, diabetes with chronic complications, drug abuse, hypertension, hypothyroidism, obesity, lymphoma, fluid and electrolyte disorders, other neurological disorders, paralysis, peripheral vascular disorders, psychoses, pulmonary circulation disorders, renal failure, ulcer disease, valvular disease, weight loss and liver disease.

**eTable 6. Association of AF with in-hospital mortality, LOS and total cost in metastatic cancer patients** **based on subgroup analysis**

| **Subgroup** | **Death** | | **LOS** | | **Cost** | |
| --- | --- | --- | --- | --- | --- | --- |
|  | **OR (95%CI)** | ***P*-value** | **Estimate** | ***P*-value** | **Estimate** | ***P*-value** |
| **Age group** |  |  |  |  |  |  |
| 18-44 | 3.58 (2.11,6.06) | <0.0001 | 0.36 | 0.0001 | 0.42 | <0.0001 |
| 45-64 | 1.98 (1.82,2.14) | <0.0001 | 0.25 | <0.0001 | 0.29 | <0.0001 |
| 65-74 | 1.52 (1.43,1.62) | <0.0001 | 0.21 | <0.0001 | 0.21 | <0.0001 |
| ≥75 | 1.38 (1.31,1.45) | <0.0001 | 0.19 | <0.0001 | 0.18 | <0.0001 |
| **Gender** |  |  |  |  |  |  |
| Female | 1.65 (1.58,1.74) | <0.0001 | 0.22 | <0.0001 | 0.21 | <0.0001 |
| Male | 1.44 (1.37,1.51) | <0.0001 | 0.17 | <0.0001 | 0.20 | <0.0001 |
| **Race** |  |  |  |  |  |  |
| White | 1.48 (1.41,1.55) | <0.0001 | 0.20 | <0.0001 | 0.20 | <0.0001 |
| Black | 1.68 (1.48,1.92) | <0.0001 | 0.14 | <0.0001 | 0.19 | <0.0001 |
| Hispanic | 1.75 (1.43,2.14) | <0.0001 | 0.18 | <0.0001 | 0.20 | <0.0001 |
| Other | 1.61 (1.34,1.94) | <0.0001 | 0.21 | <0.0001 | 0.23 | <0.0001 |
| **ECI** |  |  |  |  |  |  |
| 0 | 1.72 (1.47,2.02) | <0.0001 | 0.38 | <0.0001 | 0.40 | <0.0001 |
| 1 | 1.71 (1.56,1.87) | <0.0001 | 0.25 | <0.0001 | 0.26 | <0.0001 |
| 2 | 1.59 (1.47,1.71) | <0.0001 | 0.22 | <0.0001 | 0.22 | <0.0001 |
| ≥3 | 1.41 (1.35,1.48) | <0.0001 | 0.14 | <0.0001 | 0.16 | <0.0001 |
| **CHA2DS2-VASc** |  |  |  |  |  |  |
| <2 | 1.93 (1.78,2.10) | <0.0001 | 0.30 | <0.0001 | 0.26 | <0.0001 |
| ≥2 | 1.43 (1.38,1.49) | <0.0001 | 0.19 | <0.0001 | 0.19 | <0.0001 |
| **Prior stroke** |  |  |  |  |  |  |
| Yes | 1.24 (1.03,1.48) | 0.0238 | 0.10 | 0.0001 | 0.09 | <0.0001 |
| No | 1.54 (1.48,1.60) | <0.0001 | 0.20 | <0.0001 | 0.21 | <0.0001 |
| **Chemotherapy** |  |  |  |  |  |  |
| Yes | 1.78 (1.54,2.07) | <0.0001 | 0.18 | <0.0001 | 0.21 | <0.0001 |
| No | 1.52 (1.46,1.58) | <0.0001 | 0.20 | <0.0001 | 0.21 | <0.0001 |
| **Long-term anticoagulants** |  |  |  |  |  |  |
| Yes | 0.97 (0.83,1.14) | 0.7420 | 0.05 | 0.0052 | 0.07 | 0.0001 |
| No | 1.57 (1.51,1.63) | <0.0001 | 0.21 | <0.0001 | 0.22 | <0.0001 |
| **Major operating room procedure** |  |  |  |  |  |  |
| Yes | 1.81 (1.69,1.93) | <0.0001 | 0.24 | <0.0001 | 0.24 | <0.0001 |
| No | 1.38 (1.32,1.44) | <0.0001 | 0.13 | <0.0001 | 0.15 | <0.0001 |
| **Coronary artery disease** |  |  |  |  |  |  |
| Yes | 1.35 (1.25,1.46) | <0.0001 | 0.15 | <0.0001 | 0.15 | <0.0001 |
| No | 1.57 (1.50,1.64) | <0.0001 | 0.21 | <0.0001 | 0.22 | <0.0001 |
| **Congestive heart failure** |  |  |  |  |  |  |
| Yes | 1.14 (1.06,1.23) | 0.0004 | 0.10 | <0.0001 | 0.11 | <0.0001 |
| No | 1.65 (1.58,1.72) | <0.0001 | 0.21 | <0.0001 | 0.23 | <0.0001 |
| **Diabetes** |  |  |  |  |  |  |
| Yes | 1.38 (1.28,1.48) | <0.0001 | 0.17 | <0.0001 | 0.16 | <0.0001 |
| No | 1.57 (1.50,1.64) | <0.0001 | 0.20 | <0.0001 | 0.22 | <0.0001 |
| **Hypertension** |  |  |  |  |  |  |
| Yes | 1.52 (1.44,1.60) | <0.0001 | 0.17 | <0.0001 | 0.17 | <0.0001 |
| No | 1.53 (1.45,1.62) | <0.0001 | 0.22 | <0.0001 | 0.25 | <0.0001 |
| **Obesity** |  |  |  |  |  |  |
| Yes | 1.38 (1.14,1.67) | 0.0010 | 0.20 | <0.0001 | 0.19 | <0.0001 |
| No | 1.53 (1.47,1.59) | <0.0001 | 0.20 | <0.0001 | 0.21 | <0.0001 |
| **Renal failure** |  |  |  |  |  |  |
| Yes | 1.34 (1.21,1.48) | <0.0001 | 0.12 | <0.0001 | 0.14 | <0.0001 |
| No | 1.55 (1.49,1.61) | <0.0001 | 0.20 | <0.0001 | 0.21 | <0.0001 |
| **Valvular disease** |  |  |  |  |  |  |
| Yes | 1.26 (1.11,1.44) | 0.0005 | 0.16 | <0.0001 | 0.15 | <0.0001 |
| No | 1.55 (1.49,1.61) | <0.0001 | 0.20 | <0.0001 | 0.21 | <0.0001 |

Abbreviations: OR, odds ratio; CI, confidence interval; ECI, Elixhauser comorbidity index; LOS, length of stay

Adjusted for age, race, gender, income, insurance type, year, hospital region, hospital type, hospital bed size, primary tumor site, multiple metastatic sites (≥2), major operating room procedure, chemotherapy, long-term anticoagulants, metastatic site, coronary artery disease, prior stroke, acquired immune deficiency syndrome (AIDS), alcohol abuse, deficiency anemias, rheumatoid arthritis, chronic blood loss anemia, congestive heart failure, chronic pulmonary disease, coagulopathy, depression, uncomplicated diabetes, diabetes with chronic complications, drug abuse, hypertension, hypothyroidism, obesity, lymphoma, fluid and electrolyte disorders, other neurological disorders, paralysis, peripheral vascular disorders, psychoses, pulmonary circulation disorders, renal failure, ulcer disease, valvular disease, weight loss and liver disease

**eTable 7. Sensitivity analyses on association of AF with in-hospital mortality, LOS and total cost in metastatic cancer patients**

| **Sensitivity analysis** | **In-hospital mortality** | | **Cost** | | **LOS** | |
| --- | --- | --- | --- | --- | --- | --- |
|  | OR (95%CI) | *P*-value | Estimate | *P*-value | Estimate | *P*-value |
| Unweighted estimation | 1.48 (1.43, 1.53) | <0.0001 | 0.19 | <0.0001 | 0.17 | <0.0001 |
| Excluded patients receiving major operating room procedure | 1.38 (1.32,1.44) | <0.0001 | 0.13 | <0.0001 | 0.15 | <0.0001 |
| Doubly robust IPTW | 1.77 (1.75, 1.80) | <0.0001 | 0.24 | <0.0001 | 0.23 | <0.0001 |
| Complete case analysis | 1.48 (1.42, 1.54) | <0.0001 | 0.19 | <0.0001 | 0.18 | <0.0001 |
| Multiple imputation | 1.48 (1.43, 1.54) | <0.0001 | 0.18 | <0.0001 | 0.19 | <0.0001 |

Abbreviations: OR, odds ratio; CL, confidence interval; LOS, length of stay; IPTW, inverse probability of treatment weighting.

Adjusted for age, race, gender, income, insurance type, year, hospital region, hospital type, hospital bed size, primary tumor site, multiple metastatic sites (≥2), major operating room procedure, chemotherapy, long-term anticoagulants, metastatic site, coronary artery disease, prior stroke, acquired immune deficiency syndrome (AIDS), alcohol abuse, deficiency anemias, rheumatoid arthritis, chronic blood loss anemia, congestive heart failure, chronic pulmonary disease, coagulopathy, depression, uncomplicated diabetes, diabetes with chronic complications, drug abuse, hypertension, hypothyroidism, obesity, lymphoma, fluid and electrolyte disorders, other neurological disorders, paralysis, peripheral vascular disorders, psychoses, pulmonary circulation disorders, renal failure, ulcer disease, valvular disease, weight loss and liver disease.
